# SARS-CoV-2: The Monster Causes COVID-19

**DOI:** 10.3389/fcimb.2022.835750

**Published:** 2022-02-08

**Authors:** Chang Song, Zesong Li, Chen Li, Meiying Huang, Jianhong Liu, Qiuping Fang, Zitong Cao, Lin Zhang, Pengbo Gao, Wendi Nie, Xueyao Luo, Jianhao Kang, Shimin Xie, Jianxin Lyu, Xiao Zhu

**Affiliations:** ^1^ School of Laboratory Medicine, Hangzhou Medical College, Hangzhou, China; ^2^ Zhu’s Team, Guangdong Medical University, Zhanjiang, China; ^3^ Guangdong Provincial Key Laboratory of Systems Biology and Synthetic Biology for Urogenital Tumors, Department of Urology, The First Affiliated Hospital of Shenzhen University, Shenzhen Second People’s Hospital (Shenzhen Institute of Translational Medicine), Shenzhen, China; ^4^ Shenzhen Key Laboratory of Genitourinary Tumor, Department of Urology, The First Affiliated Hospital of Shenzhen University, Shenzhen Second People’s Hospital (Shenzhen Institute of Translational Medicine), Shenzhen, China; ^5^ Department of Biology, Chemistry, Pharmacy, Free University of Berlin, Berlin, Germany

**Keywords:** COVID-19, pandemic, prevention, *SARS-CoV-2*, therapy

## Abstract

Coronaviruses are viruses whose particles look like crowns. SARS-CoV-2 is the seventh member of the human coronavirus family to cause COVID-19 which is regarded as a once-in-a-century pandemic worldwide. It holds has the characteristics of a pandemic, which has broy -55ught many serious negative impacts to human beings. It may take time for humans to fight the pandemic. In addition to humans, SARS-CoV-2 also infects animals such as cats. This review introduces the origins, structures, pathogenic mechanisms, characteristics of transmission, detection and diagnosis, evolution and variation of SARS-CoV-2. We summarized the clinical characteristics, the strategies for treatment and prevention of COVID-19, and analyzed the problems and challenges we face.

## Introduction

**Figure 1:**
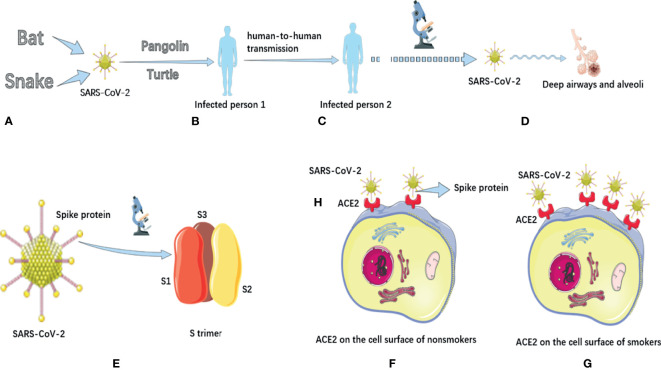
The potential animal origin, transmission characteristics, structure and pathogenic mechanisms of SARS-CoV-2. **(A)** Some studies have suggested that COVID-19 may have originated in bats or snakes. **(B)** Turtles and pangolins may be potential intermediate hosts for transmission of COVID-19 to humans. **(C)** COVID-19 has the potential for sustained human-to-human transmission and has pandemic characteristics. **(D)**. In terms of mechanism of action, COVID-19 mainly causes inflammatory responses characterized by deep airway and alveolar injury. **(E)** There are a large number of spike proteins on the surface of SARS-CoV-2. The spike protein on the surface of SARS-COV-2 is a trimer composed of three subunits with a triangular section. **(F)** Distribution of ACE2 on the cell surface of nonsmokers. **(G)** The distribution of ACE2 on the cell surface of smokers, and its expression is significantly up-regulated. **(H)** The binding receptor protein of spike protein of SARS-CoV-2 in human body is ACE2 protein. The SARS-CoV-2 has a high affinity for human ACE2.

Coronaviruses were first isolated from chickens in 1937 and are known as coronaviruses because of the coronaviruses’ crown shaped particles. Coronaviruses can cause multi-system infections in a variety of animals. Previously, there were six major types of coronavirus that can infect humans, including two highly lethal coronavirus, SARS-CoV and MERS-CoV, and four coronavirus that cause mild upper respiratory disease, namely HCoV-OC43, HCoV-229E, HCoV-NL63 and HCoV-HKU1. The pneumonia outbreak was caused by a novel coronavirus (Wu F. et al., 2020), the virus is the seventh member of the human coronavirus family (Zhu et al., 2020; Kobayashi et al., 2021). The outbreak is thought to be once-in-a-century (Gates, 2020). WHO later officially declared COVID-19 to have pandemic characteristics (**Figure 1C**). The impact of the outbreak is manifold, including the cause of scientific research (Service, 2020). The transmissibility of the virus, the full range of disease severity and the risk factors for severe illness and death are the focus of research (Lipsitch et al., 2020). In addition, COVID-19 is a zoonosis and cats are highly susceptible to the virus (Shi et al., 2020). In the study of SARS-CoV-2, proteomics and metabolomics have played an important role (Shen B. et al., 2020). In this review, the origin, structure, mechanism, characteristics of transmission, detection and diagnosis, and clinical characteristics of SARS-CoV-2, as well as the treatment and prevention, evolution and variation of SARS-CoV-2 are introduced, and the progress of related research on SARS-CoV-2 is clarified.

## The Origin of *SARS*-*CoV*-*2*

Severe acute respiratory syndrome coronavirus 2 (*SARS*-*CoV*-*2*) originated in nature (Andersen et al., 2020; Hull et al., 2021), and there are two most likely natural ways of origin: the first is that it evolved into a pathogenic state in animals and then passed to humans, and the second is that animals spread viruses that do not cause disease to humans, which then evolved into the pathogenic viruses that cause pandemic in humans. Lu et al. (2020) compared the genome sequence of SARS-CoV-2 with the virus library, and found that the genome sequence similarity of SARS-CoV-2 with two kinds of SARS-CoV-like coronavirus derived from bats was 88%, about 79% with human SARS-CoV, and only about 50% with MERS-CoV. The researchers also investigated the virus’s spike proteins, which showed that the SARS-CoV-2 had a similar structure to the human SARS-CoV. Wu A. et al. (2020) ‘s research also supports this view. Zhou et al. (2020) also pointed out that the SARS-CoV-2 belongs to the same virus species as SARS-CoV, and they indicated that this novel coronavirus may originate from bats (**Figure 1A**). Ji et al. (2020) speculated that SARS-CoV-2 might come from snakes (**Figure 1A**).

## The Structure Of *SARS*-*CoV*-*2*

Walls et al. (2020) found that the spike protein of SARS-CoV-2 has a variety of conformation states. In general, the S extracellular region of SARS-CoV-2 is a 160Å Trimer with a triangular section, similar to the closely related SARS-CoV S protein structure (**Figure 1E**). Ke et al. (2020) further revealed the high-resolution structure and distribution of trimers on the virus surface, and found that most of the spike proteins were in a pre-fusion state. In addition, on the surface of SARS-COV-2, the Receptor Binding Domain of spike protein exists in many different states. This research helps us to understand the interaction between neutralizing antibodies and viruses. Kim et al. (2020) analyzed the crystal structure of the protein Nsp15 in SARS-CoV-2, which can be used as a drug target. The structure of NSP-15 in SARS-CoV is 95% similar to that in SARS-COV-2. Previous studies have demonstrated that inhibition of Nsp15 expression can slow down the replication of SARS-CoV. This suggests that drugs that previously targeted the NSP-15 to treat SARS-CoV infection have the potential to treat COVID-19.

## Pathological Changes and the Role of ACE2 in Infection

Xu Z. et al. (2020) found that SARS-CoV-2 mainly caused an inflammatory response characterized by deep airway and alveolar injury (**Figure 1D**). However, pulmonary fibrosis and consolidation in patients with COVID-19 were not as severe as those with SARS, but the exudation response was more obvious than those with SARS. Yan et al. (2020) identified the complex structure of ACE2 full-length protein and the viral S protein receptor binding domain, which marks an important advance in understanding how SARS-CoV-2 infects human cells. Xu X. et al. (2020) pointed out that the binding receptor protein of Spike protein of SARS-CoV-2 in human body is ACE2 protein (**Figure 1H**). Wrapp et al. (2020) also pointed out that SARS-CoV-2 has a high affinity for human ACE2, which may make the virus easy to spread from person to person (**Figure 1H**). They noted that ACE2 bound to SARS-CoV-2 with approximately 10-20 times more affinity than ACE2 bound to SARS-CoV. In addition, other researchers (Ghosh et al., 2021) have found increased ACE2 expression in smokers. This increases the binding of human cells to the SARS-COV-2 S protein, making smokers more susceptible to infection (**Figures 1F, G**). Moreover, smoking can impair the body’s immune system, increasing the severity and mortality of COVID-19 patients (Gravely et al., 2021).

## The Transmission Characteristics of *SARS*-*CoV*-*2*

Chan et al. (2020) tracked the course of COVID-19 in a family of seven people and found that SARS-CoV-2 may spread from person to person without people realizing they are infected. Bai et al. (2020) reported the first case of asymptomatic infection in China. The patient had no fever and other symptoms during the incubation period, and the nose swab virus nucleic acid test was negative, but all the 5 relatives in contact with the patient were infected. The emergence of asymptomatic infections places a higher demand on the control of the pandemic. He et al. (2020) pointed out that COVID-19 patients may begin to excrete or secrete the virus before the onset of dominant symptoms, which means that the virus has been transmitted before the initial symptoms of the patients. Riou et al. (Riou and Althaus, 2020) pointed out the possibility of sustained human-to-human transmission of this virus (**Figure 1C**). Pan et al. (2020) tested stool samples from 17 confirmed patients using RT-PCR and found that 9 of them were positive. Although the viral load in these stool samples was lower than in respiratory samples, this study suggests precautions should be taken when handling stool from confirmed patients.

In terms of the intermediate host of virus transmission, Liu Z. et al. (2020) analyzed the composition and differences of SARS-CoV-2 spines protein and host ACE2 receptor, and predicted that turtles might also be the potential intermediate host for the transmission of SARS-CoV-2 to humans (**Figure 1B**). Another group of researchers (Liu et al., 2019) found coronavirus in Malayan pangolins earlier using metagenomic sequencing, suggesting that pangolins may also be a potential intermediate host for SARS-CoV-2 (**Figure 1B**). Lam et al. (2020) also pointed out that pangolin is the only known mammal other than bats and humans that has been found to carry SARS-CoV-2-like virus, which is probably one of the intermediate hosts (**Figure 1B**).

In addition, Liu Y. et al. (2020) first revealed the aerodynamic characteristics of the transmission route of SARS-CoV-2 aerosol and proposed the transmission model of viral aerosol, Guo et al. (2020) pointed out that the maximum propagation distance of SARS-CoV-2 aerosol may be 4 meters in terms of the relationship between temperature and virus transmission, Yao et al. (2020) believed that the transmission of COVID-19 was independent of climatic factors such as temperature and relative humidity, and they believed that warmer weather might not reduce the transmission of COVID-19.

## The Detection and Diagnosis of *SARS*-*CoV*-*2* Infection

PCR technology plays an important role in detection and diagnosis of COVID-19. Corman et al. (2020) found that RT-PCR technology could be used to detect SARS-CoV-2, and they also gave the sequence of detection primers and determination methods, which provided the theoretical basis and the basis for the detection of SARS-CoV-2 (**Table 1**). Barra et al. (2020) showed that the use of synthetic RT-qPCR targets to analyze novel detection and diagnostic parameters in automated workstations is a simple, rapid and effective method to deal with the threat of SARS-CoV-2 (**Table 1**). Suo et al. (2020) found that ddPCR has advantages in the detection of low-load SARS-CoV-2 clinical samples and can reduce false negatives, which may be a powerful supplement to the existing standard RT-PCR (**Table 1**).

**Table 1:** The diagnosis and detection of SARS-CoV-2 and COVID-19.

Subjects that are based on	Methods	Advantages	References
Medical imaging	An artificial intelligence model for rapid diagnosis using CT images	The total accuracy of diagnosis of COVID-19 and other viral pneumonia was 83%.	Wang et al. (2021) Eur Radiol
Molecular biology	RT-PCR	This technique detects a wide range of viruses with high sensitivity.	Corman et al. (2020) Euro Surveill
	COVID-19 nucleic acid detection paper using SHERLOCK technology based on CRISPR system.	The test can complete the diagnosis in less than an hour.	Kellner et al. (2019) Nat Protoc
	Detection of Nucleocapsid Protein	This detection method’s repeatability is good and sensitivity is high.	Sun et al. (2022) Bioengineered
	Modified COVID-19 detection kit	This method can be used to detect samples with a low viral load.	Itokawa et al. (2020) PLoS One
	RT-qPCR	This is a simple, quick and effective way to deal with the COVID 19 threat.	Barra et al. (2020) Genes (Basel)
	ddPCR	This method has advantages in the detection of low-load COVID-19 clinical samples and can reduce false negatives.	Suo et al. (2020) Emerg Microbes Infect

In addition, Wang et al. (2021) developed an artificial intelligence model that used CT images to assist the rapid diagnosis of SARS-CoV-2, and the total accuracy of this model in the diagnosis of SARS-CoV-2 and other viral pneumonia reached 83% (**Table 1**). Wu et al. (Wu and McGoogan, 2020) summarized the clinical reports of 72,314 patients and pointed out that the diagnosis method of SARS-CoV-2 was nucleic acid detection of respiratory tract samples, but clinical diagnosis could also be made according to exposure history, symptoms and chest imaging. Other researchers have utilized the SHERLOCK technology based on the CRISPR system (Kellner et al., 2019) to launch a SARS-CoV-2 nucleic acid test paper, which can make a diagnosis in less than an hour (**Table 1**). Sun et al. (2022) has found a rapid method to detect the N protein of the virus. Their detection method’s repeatability is good and sensitivity is high (**Table 1**). Itokawa et al. (2020) replaced a primer in the SARS-CoV-2 detection kit, thereby expanding the lower limit of genomic analysis of the entire SARS-CoV-2 to detect lower viral load samples (**Table 1**). This contributes to a deeper understanding of the genomic epidemiology of this pathogen.

## The Clinical Features of COVID-19

Backer et al. (2020) estimated that the incubation period of this virus ranged from 1 day to more than 11 days, averaging about 6 days. Guan et al. (2020) pointed out that the median incubation period of COVID-19 is 3 days, and the longest incubation period can be up to 24 days. The most common symptoms of patients are fever and cough. Wang D. et al. (2020) described the clinical characteristics of 138 patients infected with SARS-CoV-2. Symptoms also contain fever, dry cough, difficulty breathing and fatigue. In addition, Sancho Ferrando (Sancho Ferrando et al., 2022) et al. found that patients with severe COVID-19 were more likely to develop acute kidney injury. Liu L. et al. (2020) showed that, without differentiating the detection time, antibody was detected in serum of 81.5% of COVID-19 patients, which was far higher than the overall positive rate of nucleic acid detection of 64.3%. Another group of researchers (Liu J. et al., 2020) found that the NLR was an independent risk factor for patients with COVID-19 to develop severe illness. Patients with age ≥50 years, NLR≥3.13 and worsening should be admitted to intensive care unit as soon as necessary. Gao et al. (2020) pointed out that patients with NT-proBNP level higher than 88.64pg/mL had a greater risk of in-hospital mortality, which could be used as an independent risk factor for in-hospital mortality in patients with severe COVID-19. In terms of cardiovascular diseases, Zeng et al. (2020) reported the first case of COVID-19 infection with myocarditis complications, and their results showed that patients with COVID-19 may develop severe cardiac complications such as myocarditis and heart failure.

## The Treatment and Prevention of COVID-19

As early as 2016, Zumla et al. (2016) explored available strategies and potential targets for the development of anti-coronavirus drugs. Regarding this outbreak, Li et al. (Li and De Clercq, 2020) pointed out that no drug or vaccine has been approved for the prevention and treatment of COVID-19, but there are a variety of schemes that are expected to control or prevent the infection, including monoclonal antibodies and drugs based on oligonucleotides (**Table S1**). Wrapp et al. (2020) successfully constructed the first 3D atomic level map of the S protein trimer on the virus surface using cryo-electron microscopy technology, which will be helpful for the development of vaccines and antiviral drugs. Holshue et al. (2020) reported that, based on the deteriorating clinical status of the patient, the decision was made to give remdesivir and received good results. This study suggests that remdesivir may be a potential wonder drug for the COVID-19 (**Table S1**). Wang M. et al. (2020) also pointed out that remdesivir and chloroquine phosphate had inhibitory effects on COVID-19 (**Table S1**). Li et al. (Runfeng et al., 2020) pointed out that the traditional Chinese medicine Lianhuaqingwen could significantly inhibit the replication of SARS-CoV-2 in cells, and after the treatment of Lianhuaqingwen, the expression of cytokines induced by virus particles was significantly reduced (**Table S1**). Based on Zhang et al.’s research (Zhang et al., 2019), Ifenprodil is considered as a potential first-line drug for the treatment of COVID–19 (**Table S1**). Duan et al. (2020) showed that plasma therapy may provide a useful therapeutic effect and a low risk in the treatment of severe COVID - 19 patients (**Table S1**). In addition, Russell et al. (2020) point out that there is no evidence to expect patients to benefit from corticosteroid therapy, and instead patients may suffer from such therapy.

In terms of prevention of COVID-19, Ahmed et al. (2020) identified a group of potential targets for SARS-CoV-2 vaccines, pointing out key experimental directions for the development of a vaccine for pneumonia caused by SARS-CoV-2. Kafader et al. (2020) proposed a method that can fundamentally detect the detailed information of a single protein molecule or its ligand, which will be helpful for the development of SARS-CoV-2 vaccine. Yang et al. (2020) pointed out that recombinant RBD protein vaccine was an important direction of SARS-COV-2 vaccine. van Doremalen et al. (2020) and Mercado et al. (2020) proposed the idea of adenovirus vaccine. So far, several types of SARS-COV-2 vaccines have been used around the world. And these vaccines have become a powerful weapon in the fight against the pandemic. Vaccination protects not only those who are vaccinated, but also those who are not (Milman et al., 2021). Moreover, the safety of sarS-COV-2 vaccine is assured (Barda et al., 2021). In addition, Ong et al. (2020) reported the distribution of the virus in the ward environment, which is of guiding significance to the cleanliness of the patient’s ward and residence, and the protection of the patient’s family members and medical staff. Nicolaides et al. (2019) pointed out that increasing hand-washing rates at the top 10 airports in the world could significantly reduce the spread of many infectious diseases, and the study suggested that personal protective measures such as frequent hand-washing could help fight the pneumonia outbreak (**Table S1**). van Doremalen et al. (2020) showed that SARS-CoV-2 could survive for up to 2-3 days in the public environment, indicating the importance of cleaning and sterilizing various solid surfaces to prevent virus transmission (**Table S1**).

## The Evolution and Variation of *SARS*-*CoV*-*2*

Lv et al. (2020) confirmed that novel coronavirus evolves under relatively strict selective pressure, and their discovery may help researchers to understand the special evolutionary mechanism that induces virus pathogenicity and how the virus adapts to the host. Shen Z. et al. (2020) found elevated levels of viral diversity in some infected individuals, suggesting a risk of rapid evolution. Although no evidence of intra - host mutation transmission has been found, this risk should not be ignored (Emrani et al., 2021; Xu et al., 2021; Zhang et al., 2021a). So far, SARS-COV-2 has been identified as Alpha, Beta, Gamma, Zeta, Delta, Lambda, Omicron and other variants, two of which need to be noted. The Delta variant of the virus spreads faster and infects more cells because it catalyzes the fusion process between virus particles and human cell membranes, allowing them to enter cells more efficiently (Zhang et al., 2021b). The Omicron variant of the virus is highly resistant to human immune serum, and there is an escape from human immunity, though fortunately this escape is not complete (Wang et al., 2022).

## Conclusions and Challenges

SARS-CoV-2 is the seventh member of the human coronavirus family, which caused a serious global pandemic, raised a great threat to human health, seriously affected the normal order of social life, is a veritable “trouble maker”. This review introduces SARS-CoV-2 from the aspects of origin, structure, mechanism of action, transmission characteristics, detection and diagnosis, relevant clinical characteristics, treatment and prevention, evolution and variation, and summarizes the characteristics and laws of this “trouble maker” from different perspectives. Humans have made significant efforts in the fight against COVID-19 and have achieved some results, including finishing the task of developing vaccines (Fathizadeh et al., 2021) and specific drugs (Shamsi et al., 2021). But we still face the threat of “re-emergence” of the virus. Despite the many challenges ahead, through the concerted efforts of all mankind, we will finally overcome this virus.

## Author Contributions

XZ and JLy conceived the work. CS wrote and drafted the manuscript. ZL, CL, MH, Jli, QF, ZC, LZ, PG, WN, XL, JK, SX, and XZ discussed and editing the manuscript. All authors read and approved the final version of the manuscript.

## Funding

This work was supported partly by National Natural Science Foundation of China (81972366); Guangdong Key Laboratory funds of Systems Biology and Synthetic Biology for Urogenital Tumors (2017B030301015) and its Open Grant (2021B030301015-3).

## Conflict of Interest

The authors declare that the research was conducted in the absence of any commercial or financial relationships that could be construed as a potential conflict of interest.

## Publisher’s Note

All claims expressed in this article are solely those of the authors and do not necessarily represent those of their affiliated organizations, or those of the publisher, the editors and the reviewers. Any product that may be evaluated in this article, or claim that may be made by its manufacturer, is not guaranteed or endorsed by the publisher.

## Abbreviations

3D, three dimensional; ACE2, angiotensin converting enzyme 2; ADARs, Adenosine deaminases acting on RNA; APOBEC, Apolipoprotein B mRNA-editing enzyme catalytic polypeptide; COVID-19, Corona Virus Disease 2019; CT, computed tomography; CRISPR, Clustered regularly interspaced short palindromic repeats; ddPCR, Droplet Digital PCR; HCoV-OC43, Human coronavirus OC43; HCoV-229E, Human coronavirus 229E; HCoV-NL63, Human coronavirus NL63; HCoV-HKU1, Human coronavirus HKU1; I-TASSER, Iterative Threading Assembly Refinement; MERS-CoV, Middle East Respiratory Syndrome Coronavirus; NLR, Neutrophil-to-Lymphocyte Ratio; NT-proBNP, N terminal pro B type natriuretic peptide; RNA, ribonucleic acid; RT-PCR, Reverse Transcription-Polymerase Chain Reaction; RT-qPCR, Quantitative reverse transcription PCR; SARS, severe acute respiratory syndrome; SARS-CoV, Severe acute respiratory syndrome coronavirus; SHERLOCK, specific high-sensitivity enzymatic reporter unlocking; TMPRSS2, Transmembrane protease serine 2; WHO, World Health Organization.
