# Supplementary material for: SARS-CoV-2: The Monster Causes COVID-19
Source: Front Cell Infect Microbiol. 2022 Feb 8;12:835750. doi: 10.3389/fcimb.2022.835750 (PMC8861077; doi:10.3389/fcimb.2022.835750)
Supplement: Supplementary file 1 [file Table_1.doc]

**Table S1. The treatment and prevention of COVID-19**

| Medicine and method | Significance | References |
| --- | --- | --- |
| Monoclonal antibodies and drugs based on oligonucleotides | They are expected to control or prevent the infection. | Li et al. (2020) Nat Rev Drug Discov |
| Remdesivir | It has a good effect on patients with deteriorating clinical status. | Holshue et al. (2020) N Engl J Med |
| Chloroquine phosphate | This drug has an inhibitory effect on COVID-19. | Wang et al. (2020) Cell Res |
| Lianhuaqingwen | It significantly inhibited COVID-19 replication in cells. | Li et al. (2020) Pharmacol Res |
| Ifenprodil | It is considered a potential first-line treatment for COVID-19. | Zhang et al. (2020) mSystems |
| Convalescent plasma | In the treatment of severe COVID-19 patients, plasma therapy may provide a favorable therapeutic outcome with a low risk. | Duan et al. (2020) Proc Natl Acad Sci U S A |
| Taking personal protective measures such as frequent hand washing | This can significantly reduce the spread of many infectious diseases. | Nicolaides et al. (2020) Risk Anal |
| Cleaning and disinfecting various solid surfaces | This measure is important to prevent the spread of the virus. | van Doremalen et al. (2020) N Engl J Med |
